# Pit viper envenomation in pediatric dogs: 5 cases

**DOI:** 10.1002/ccr3.5065

**Published:** 2021-11-12

**Authors:** Carl J. Southern, Ashley E. Allen‐Durrance, Michael Schaer

**Affiliations:** ^1^ College of Veterinary Medicine Small Animal Clinical Sciences University of Florida Gainesville Florida USA

**Keywords:** antivenom, dog, pit viper, puppy, snake

## Abstract

Treatment of pit viper envenomated puppies (≤6  months old) with antivenom was well tolerated, similar to adult dogs. However, therapeutic guidelines should be established to direct use and prove efficacy in this population.

## Graphical abstract

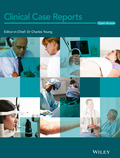

## 1 INTRODUCTION

A young age should not discourage early and appropriate use of antivenom in puppies envenomated by pit vipers, following the same treatment guidelines as those established for adult dogs. This case series reports a successful outcome in five pit viper envenomated puppies treated with antivenom.

Snakebite envenomation is a common medical emergency in several regions of the United States. North central Florida is home to several indigenous pit vipers including the eastern diamondback rattlesnake (*Crotalus adamanteus*), the water moccasin (*Agkistrodon piscivorus*), timber rattlesnake (*Crotalus horridus*), and the pygmy rattlesnake (*Sistrurus miliarius*).^1^ The current canine veterinary literature describes snakebite envenomation in adults, excluding the effects or outcomes on pediatric patients.

Many dogs free roam leading to several unwitnessed bites. Therefore, the attending clinician often makes the diagnosis based on history, physical examination, and supporting characteristic effects of envenomation including coagulopathy, hemolysis, neurological signs, hypotension, pain, and soft tissue damage. Clinical signs vary according to the species of snake involved, amount of venom delivered, venom components in the particular snake, and previous comorbidities of the victim. Most pit viper venom causes various combinations of hemolysis, vasculotoxicity, tissue toxicity, coagulopathy, and rhabdomyolysis. Pygmy rattlesnake venom causes pain at the bite site and thrombocytopenia. Cardiotoxicity and neurotoxicity are additional features of eastern diamondback rattlesnake venom while some timber rattlesnakes have potent neurotoxicity.^2^

Limited evidence‐based data exist to guide medical management of pit viper envenomation in children, and no pediatric guidelines exist in veterinary medicine. Historically, there were increased concerns for pit viper envenomation in children due to smaller patient size, theoretically exposing the child to a higher concentration of venom per kilogram and potentially more severe consequences from the venom compared to adults.^3^ Recent literature in human medicine advocates for judicious use of antivenom based on systemic involvement (coagulopathy, shock, neurological signs, gastrointestinal signs, etc.) with infrequent fasciotomies reserved for management of compartment syndrome.^4^, ^5^ Although variable, antivenom protocols in children recommend initial control of systemic signs with 4–18 (mean 7.7) vials of F(ab) antivenom with subsequent maintenance therapy of 2 vials every 6 h.^6^ Hospitalization ranges from 1 to 8 days (average 2 days), use of empirical antimicrobial therapy is discouraged, and the outcome is often favorable.^5^, ^7^, ^8^

The objectives of this case series are to describe pit viper envenomation in five pediatric dogs, which has not been previously reported, and to demonstrate that treatment conforms to standard treatment recommended for mature dogs.

## 2 MATERIALS AND METHODS

Five puppies <6 months of age presented to an academic veterinary teaching hospital for either a witnessed or suspected pit viper bite between 2013 and 2018. Two veterinary scoring systems, the Colorado acute pain score and the snakebite severity score (SSS), were used to assess pain and envenomation signs to guide therapeutic intervention. The Colorado acute pain score assigns pain from 0 to 4 and is used in combination with the SSS to guide the use of additional antivenom and/or analgesics. The SSS assigns a score of 0–3 or 4 to six body areas affected by pit viper envenomation: pulmonary system, cardiovascular system, local wound, gastrointestinal system, hematological system, and central nervous system. A score of 0 is consistent with no signs of envenomation whereas a score of 20 is consistent with severe envenomation. This scoring system is most useful for monitoring trends in pit viper envenomated animals and can be misleading in the subacute setting as signs can be delayed. In addition to serial physical examinations and SSS assessment, point‐of‐care diagnostic included cytological blood smear review, activated clotting time, packed cell volume, total protein, and venous blood gas. All puppies received at least one vial of F(ab)_2_ antivenom (VenomVet^®^, MT Venom, LLC) at the discretion of the treating clinician. At the author's institution, both F(ab)_2_ and whole IgG equine‐derived pit viper antivenoms are available; whereas, F(ab) ovine‐derived pit viper antivenom commonly used in human medicine is cost‐prohibitive in most veterinary patients. The F(ab)_2_ product is potentially less antigenic leading to fewer adverse reactions and may penetrate tissues better due to smaller molecular size, therefore, it is routinely used first and further use is based on serial monitoring. Additional therapies included fluid therapy, analgesics if pain was not adequately controlled with antivenom alone, and antibiotics when indicated.

## 3 CASE 1

A 14 week‐old, 10.8 kg male intact American Pit Bull Terrier was evaluated for suspect pit viper envenomation. The puppy was unsupervised outside for 2 h and found limping on his left hindlimb with two bleeding punctures over his fourth digit. Physical examination revealed left pelvic limb weight‐bearing lameness with hemorrhagic lymphedema around two punctures on the left hind paw. The mentation and vitals were normal. A pain score (Colorado State University Acute Pain Scale) of 1/4 was assigned. Clinicopathologic abnormalities included: mild hyponatremia (140 mmol/L, reference range 146–151 mmol/L), mild hypochloremia (105 mmol/L, reference range 108.5–116 mmol/L), mild anemia [packed cell volume (PCV) 32%, reference range 33–55%] without hemolysis, and moderate hypoproteinemia [total solids (TS): 48 g/L (reference range 65–80 g/L); 4.8 g/dL (reference 6.5–8 g/dL)]. The activated clotting time (ACT) was normal (96 s (s); reference range 80–120 s). A snakebite severity score (SSS)^9^ was 1/20. One vial of F(ab)_2_ antivenom (VenomVet^®^, MT Venom, LLC) was diluted in 60 mL 0.9% saline (0.9% sodium chloride, Abbott Laboratories) and administered intravenously (IV) over an hour as the potential envenomation occurred within 30 min to 2 h prior to presentation and there was concern for progression in the subacute setting. Six hours later, the SSS was 2/20. The puppy was maintained on IV lactated Ringer's solution (LRS) (Lactated Ringer's Solution, Baxter Laboratories) at 4 mL/kg/h and methadone (Methadone hydrochloride, Mallinckrodt Inc) (0.25 mg/kg IV) when pain scores exceeded 1.5/4. No progression of envenomation or adverse reactions to antivenom occurred and the puppy was discharged 20 h later.

## 4 CASE 2

An 11‐week‐old, 7.4 kg female intact mixed‐breed dog was presented for suspected pit viper envenomation. While outside, the puppy cried out, became lame on the left forelimb, and was evaluated within 40 min. The temperature, heart, and respiratory rates were 39°C (102.3°F), 280 beats/min, and 80 breaths/min, respectively. The puppy was laterally recumbent, obtunded, and had weak pulses despite a systolic blood pressure of 150 mm Hg. Severe swelling with two actively bleeding puncture wounds and ecchymoses were found on the left shoulder. The pain score (Colorado State University Acute Pain Scale) was 4/4. Clinicopathologic abnormalities included: mild hyperlactatemia (2.5 mmol/L, reference range 0.4–1.5 mmol/L), anemia (PCV 27%, reference range 33–55%), and severe thrombocytopenia (0–1 platelet/HPF with no clumping, reference range >10/HPF), and hypocoagulability (ACT 208 s, reference range 80–120 s). The SSS was 10/20. Two 15 mL (2 mL/kg) IV boluses of hypertonic saline (Vet One, MWI Animal Health) were administered followed by a 20 mL/kg IV bolus then 3 mL/kg/h IV LRS (Lactated Ringer's Solution, Baxter Laboratories). One vial of F(ab)_2_ antivenom (VenomVet^®^, MT Venom, LLC) was diluted in 60 mL 0.9% saline (0.9% sodium chloride, Abbott Laboratories) and administered IV over 20 min. An hour later, a repeat ACT was >999s (reference range 80–120 s) and the SSS increased to 12/20. Two additional vials of diluted F(ab)_2_ antivenom (VenomVet^®^, MT Venom, LLC) were administered IV over an hour. Three hours post‐presentation, the SSS remained 10/20 and the ACT was >999s (reference range 80–120 s); therefore, a fourth vial of diluted F(ab)_2_ antivenom (VenomVet^®^, MT Venom, LLC) was administered IV over an hour. The puppy remained tachycardic (230–270 beats/min) and normotensive; therefore, 0.2 mg/kg methadone (Methadone hydrochloride, Mallinckrodt Inc) IV was administered. Five hours post‐presentation, the PCV/TS was 20% (reference range 33–55%) and 34 g/L (reference range 65–80 g/L; 3.4 g/dL, reference range 6.5–8 g/dL), respectively. Two additional vials of diluted F(ab)_2_ antivenom (VenomVet^®^, MT Venom, LLC) were administered. Approximately 6 h post‐presentation and after six vials of antivenom, the SSS decreased to 9/20 and mentation improved.

Thirteen hours post‐presentation, the skin proximal to the bite site blackened. The puppy remained tachycardic (260 beats/min) with strong pulses, mucus membranes were pale, and non‐localizing pain was evident on abdominal palpation. Repeat clinicopathologic parameters showed anemia [HCT 17%, reference range 33–55%; hemoglobin 55 g/L, (5.5 g/dL); reference range 140–260 g/L (14–26 g/dL)], hypokalemia (3.73 mmol/L; reference range 3.98–4.41 mmol/L), hyponatremia (136.7 mmol/L; reference range 146–151 mmol/L), and mild hyperlactatemia (2.3 mmol/L; reference range (0.4–1.5 mmol/L)). Hypocoagulability resolved (ACT 74 s; reference range 80–120 s) and the puppy was hypertensive at 170 mm Hg. An IV fentanyl (Fentanyl citrate, Akorn, Inc) constant rate infusion (CRI) at 2 µg/kg/h and ampicillin sulbactam (Unasyn^®^, Pfizer) 30 mg/kg IV q8h were begun. Three vials of diluted F(ab)_2_ antivenom (VenomVet^®^, MT Venom, LLC) were administered IV as a CRI over 4 h. The SSS 24 h after admission was 6/20. The puppy began to eat, and the antibiotic was transitioned to amoxicillin/clavulanic acid (Clavamox^®^, Zoetis Inc) 125 mg (16.8 mg/kg) PO q12h. Repeat PCV/TS were 13% (reference range 33–55%) and 40 g/L (4 g/dL) [reference range 65–80 g/L (6.5–8 g/dL)], respectively. A packed red blood cell transfusion (15.4 mL/kg) was administered over 4 h. Two hours post‐transfusion the PCV/TS were 23% (reference range 33–55%) and 42 g/L (4.2 g/dL) [reference range 65–80 g/L (6.5–8 g/dL)], respectively. Fentanyl (Fentanyl citrate, Akorn, Inc) CRI was discontinued, and tramadol (Tramadol 50 mg tabs, Jannssen Ortho LLC, Puerto Rico) was started at 25 mg (3.4 mg/kg) PO q12h. The puppy received one 45‐min hyperbaric oxygen chamber treatment prior to discharge. By Day 4, the bite wound had well‐demarcated margins but did not progress to sloughing.

## 5 CASE 3

A 15 week‐old, 9.1 kg female intact Golden Retriever puppy was examined within 30 min of suspected pit viper envenomation after being found outside with a left swollen muzzle and two puncture wounds two centimeters apart. Aside from moderate left‐sided facial swelling, the physical examination was unremarkable. Vitals were normal and the pain score (Colorado State University Acute Pain Scale) was 0/4. Abnormal clinicopathologic parameters included mild hypoproteinemia [TS 50 g/L (5 g/dL), reference range 65–80 g/L 6.5–8 g/dL] with slight hemolysis and hypocoagulability (ACT >999 s; reference range 80–120 s). The SSS was 6/20 and one vial of diluted F(ab)_2_ antivenom (VenomVet^®^, MT Venom, LLC) was administered IV over 4 h, followed by IV LRS (Lactated Ringer's Solution, Baxter Laboratories) at 3 mL/kg/h. Six hours post‐presentation, the ACT remained too high to read and the SSS was 6/20. A second vial of diluted F(ab)_2_ antivenom (VenomVet^®^, MT Venom, LLC) was administered IV over 4 h. The puppy was hospitalized overnight where coagulopathy resolved and clinical improvement occurred with lower SSS values of 2/20 and 1/20 at 13 h and 25 h after admission, respectively. By day two, the puppy was clinically stable and discharged.

## 6 CASE 4

A 12‐week‐old, 4.3 kg male intact Beagle presented 1 h after a witnessed pygmy rattlesnake bite to the muzzle. There was a noticeable swelling localized to the muzzle that was sensitive to touch and two visible bite wounds ventral to the nares. Vitals were normal. Abnormal clinicopathologic parameters included mild hyponatremia (140.5 mmol/L; reference range 146–151 mmol/L). The PCV and TS were 46% (reference range 33–55%) and 58 g/L (5.8 g/dL) [reference range 65–80 g/L (6.5–8 g/dL)] with no hemolysis, respectively; and, the ACT was 81 s (reference range 80–120 s). The puppy's initial SSS was 2/20. One vial of F(ab)_2_ antivenom (VenomVet^®^, MT Venom, LLC) was diluted in 100 mL 0.9% saline (0.9% sodium chloride, Abbott Laboratories) with 25 mL administered as a bolus, then 75 mL administered as a CRI over 6 h. The puppy was maintained on IV LRS (Lactated Ringer's Solution, Baxter Laboratories) at 3 mL/kg/h. Six hours later, the ACT was normal and SSS was 2/20. The facial swelling spread to the face, lips, and eyelids. The puppy was discharged after 24 h.

## 7 CASE 5

A 20‐week‐old, 15.2 kg female intact Australian Blue Heeler was presented after an unwitnessed bite 30 min before arrival. Physical examination revealed moderate right‐sided muzzle swelling with two bleeding puncture wounds ventral to the right eye. The puppy appeared dull, nauseous, and had a tense abdomen. The puppy's pain score (Colorado State University Acute Pain Scale) was 2/4, and vitals were normal. Abnormal clinicopathologic parameters included mild hyperlactatemia (2.4 mmol/L; reference range 0.4–1.5 mmol/L) and hemolyzed serum with a normal PCV and TS, 53% (reference range 33–55%) and 78 g/L (7.8 g/dL) [reference range 65–80 g/L (6.5–8.0 g/dL)], respectively. Blood smear revealed echinocytosis and mild thrombocytopenia (mean 9 platelets per HPF; reference range >10 platelets/HPF). Systolic blood pressure was 90 mm Hg, and the ACT was 58 seconds (reference range 80–120 s). The SSS was 5/20. The puppy was administered one vial of diluted F(ab)_2_ antivenom (VenomVet^®^, MT Venom, LLC) IV over 30 min and a second vial over the next 2 h along with LRS (Lactated Ringer's Solution, Baxter Laboratories) IV fluids at 3 mL/kg/h. Six hours later, the SSS remained 5/20. The puppy was hospitalized overnight for monitoring. The facial swelling continued to spread, but the pain resolved with antivenom therapy. By day two, the appetite was hearty, the SSS had decreased to 2/20, and the puppy was discharged.

## 8 DISCUSSION

North central Florida is a unique geographic area home to five indigenous venomous pit vipers including water moccasins, and eastern diamondback, timber, and pygmy rattlesnakes. The amount of venom released varies according to the species, snake size, time lapse since the last venom delivery, and degree of threat sensed before venom delivery.^10^, ^11^, ^12^ Adult dogs might pose more of a threat compared to the playful and naive nature of puppies, which would cause the snake to release more venom into the adult victim.^10^ This might be a contributing reason to explain why all five puppies in this series survived.

All five of the puppies were taken to the emergency hospital within 2 h following their likely snake encounter. Four of these were fortunate to maintain stable vital signs upon presentation. Case two was on the threshold of decompensation, but readily stabilized with emergency treatment. In envenomated children, their small size was traditionally thought to predispose them to a greater amount of venom per square meter area, thus exposing more cells to the adverse effects of the venom.^13^, ^14^ In Parish's study dating as far back as 1965, the majority of children did well as long as they received antivenom.^13^ In LoVecchio's study of 66 envenomated children over 10 years, 38% of children developed a hypersensitivity to whole IgG antivenom with five cases of morbidity, but all survived.^14^ These reports of increased rather than decreased survival are likely due to the availability of antivenom and its use in children during the 1960’s and after. The same guarded prognosis was assumed with this young canine population, but all puppies in this report survived as well with timely antivenom and medical management.

These five puppies are the first cluster of canine pediatric snakebite victims, aged 11–20 weeks old, in the United States to be described. The clinical signs in this particular group were similar to those seen in many adult dogs in the same region.^15^, ^16^ Two of the four had prolonged activated clotting times, with bleeding restricted to the bite site. The ACT was selected to monitor coagulopathy because of the small amount of blood required for this test, its relatively low cost, the proximity of the machine to the emergency room, and its proven clinical benefits as a point‐of‐care test when managing snakebite victims with coagulopathies.^17^

Three of the five puppies were bitten in the face while two were bitten in the limbs which is a similar presentation to that seen in adult dogs.^15^ The snakebite severity scores ranged from 1 to 10 with all values reverting to minor status by day two following treatment.

The amount of antivenom administered to each puppy paralleled its SSS whereby those with scores <2 receiving 1–2 vials, and the most severely affected puppy (case two) with a presenting score of 10 received proportionally more antivenom (nine total vials). None of the puppies in this case series developed a hypersensitivity reaction to the equine‐derived F(ab)_2_ antivenom (VenomVet^®^, MT Venom, LLC). In children, hypersensitivity reactions to equine‐derived IgG antivenom are common (38%),^14^ while less common (2.8%)^7^ when administered ovine‐derived F(ab) antivenom. As this is a small case series, it is likely that equine‐derived F(ab)_2_ antivenom has the potential to cause hypersensitivity reactions in puppies, similar to adult dogs.^15^

Fluid overload in children weighing less than 10 kg and those with physiologic concerns for fluid overload, such as congestive heart failure, chronic lung disease, and renal insufficiency is a concern in human medicine when multiple doses of antivenom are administered as their protocols typically dilute antivenom in 250 mL 0.9% saline.^7^ Puppies in this study ranged from 4.3 to 15.2 kilograms, and the same risk for fluid overload should be considered when treating pediatric dogs requiring multiple doses of antivenom. In this series, antivenom was diluted in 50–100 mL 0.9% saline at the clinician's discretion, and no reported complications from fluid overload occurred.

The small number of puppies included in this case series limits statistical analysis to determine prognostic factors. Due to the retrospective nature of this case series and medical record system at the author's institution, cases were obtained by searching invoice items for pit viper antivenom and age of 6 months or less in dogs. As such, puppies presenting to the emergency room with a snakebite, but did not receive antivenom, due to lack of clinical need or owner financial restrictions, would have been overlooked in data collection. In addition, the use of antivenom may not be necessary for all patients. For example, some clinicians may have elected to withhold antivenom from case 1 based on minimal signs of envenomation. In a subacute setting, antivenom could be withheld from an animal with a high venom load based on the SSS to later have that animal show severe signs of envenomation and be more at risk for sequelae from envenomation such as bleeding, anemia, and acute kidney injury. This clinical conundrum may lead to the overuse of pit viper antivenom in veterinary medicine. Until more objective data such as a bedside diagnostic test to determine venom load exists, overuse of antivenom may or may not occur. Future studies should enroll pediatric patients prospectively for data collection and include measurement of venom concentration in serum and/or urine to confirm diagnosis and guide evidence‐based medicine.

## CONFLICT OF INTEREST

None.

## AUTHOR CONTRIBUTIONS

Dr. Southern was primary author and prepared the first draft. All authors contributed equally to conception, design, analysis, interpretation, and revisions after submission. Drs. Allen‐Durrance and Schaer contributed equally to critical revisions prior to submission. Dr. Allen‐Durrance was senior and corresponding author.

## ETHICAL APPROVAL

The authors confirm all patients were treated with current standard of care in veterinary medicine. Due to the retrospective nature of this case series, approval by a licensing board was not required.

## CONSENT

The authors confirm that client consent has been signed and collected in accordance with the journal's client consent policy.

## Data Availability

Data sharing not applicable to this article as no datasets were generated or analyzed during the current study.
